# Changes induced by diet and nutritional intake in the lipid profile of female professional volleyball players after 11 weeks of training

**DOI:** 10.1186/1550-2783-10-55

**Published:** 2013-12-11

**Authors:** Juan Mielgo-Ayuso, Pilar S Collado, Aritz Urdampilleta, José Miguel Martínez-Sanz, Jesús Seco

**Affiliations:** 1 Department of Nutrition and Dietetics, Haro Volleyball Club, Nutrition Centre of La Rioja, C/ Donantes de sangre, 14.26200 Haro, La Rioja, Spain; 2 Department of Biomedical Sciences, University of Leon, Leon, Spain; 3 Institute of Biomedicine (IBIOMED), University of Leon, Leon, Spain; 4 Public Sports Education Center, Kirolene, Basque Government, Scientific-Technical Planning for Sports, Nutriaktive, Vitoria-Gasteiz, Spain; 5 Department of Nursing, Faculty of Health Sciences, University of Alicante, Alicante, Spain; 6 Visiting Researcher at the University of the Basque Country, Leioa, Bizkaia, Spain

**Keywords:** Lipids, Atherogenic indices, Sports nutrition, Team sports, Elite athletes, Dietary intake, Female athletes

## Abstract

**Background:**

The relationship between cardiovascular disease and lipid profile is well known. Apart from a heart-healthy diet, exercise is the primary factor that can modify this lipid-associated cardiovascular risk. The aim of the study was to evaluate potential changes in the levels of triglycerides, total cholesterol (TC), low-density lipoprotein-cholesterol (LDLc), and high-density lipoprotein-cholesterol (HDLc), as well as atherogenic indices (TC/HDLc and LDLc/HDLc), and also to analyse the diet over 11 weeks of training in female professional volleyball players.

**Methods:**

The lipid profile of 22 female professional volleyball players was analysed on Day T0 (pre-preseason) and Day T11 (after 11 weeks of training). The consumption of fats by the players was estimated using a food frequency questionnaire, confirmed by seven days of full dietary records.

**Results:**

By the end of the study, the LDLc levels and both atherogenic indices of the players had decreased (p < 0.05) compared to the values obtained at baseline. In addition, the diet of the players contained 35.5 ± 3.2% of fats (saturated fatty acid: 11.1 ± 1.2%, monounsaturated fatty acid: 14.3 ± 1.9%, and polyunsaturated fatty acid: 7.0 ± 1.1%) and 465 ± 57 mg of dietary cholesterol. Their score for the (monounsaturated + polyunsaturated fatty acid)/saturated fatty acid ratio was 1.9 ± 0.4, less than the recommended ≥ 2.

**Conclusion:**

These data indicate that the activity of the female professional volleyball players during the first 11 weeks of training in the season was heart healthy, because their lipid profile improved, despite an inadequate intake of fats.

## Background

Physical activity and a heart-healthy diet, such as the Mediterranean diet [1], have been highlighted as major factors in preventing cardiovascular disease (CVD) [2]. Therapeutic lifestyle changes, including nutrition and exercise, are recommended as the front-line strategy for addressing cardiovascular risk factors. Moreover, the positive relationship between CVD and concentrations of low-density lipoprotein cholesterol (LDLc) and the negative relationship between concentrations of high-density lipoprotein cholesterol (HDLc) and cardiovascular risk have been clearly established in numerous clinical trials [3]. Extensive physical activity is one of the factors that have been shown to be associated with high concentrations of HDLc, which may in part explain the lower risk of coronary heart disease in physically active people [4]. Furthermore, the influence of diet on plasma lipid levels is well known, in particular, the fact that the impact on cardiovascular risk is dependent on the saturated or unsaturated nature, as well as on the number of carbon atoms in the chain, of the fatty acids consumed [5]. In a recent meta-analysis, Kelley et al. [6] concluded that a proper diet along with a programme of aerobic exercise (brisk walking, swimming, cycling, aerobics, or racquet sports) improved the lipid profile (LP), thanks to decreased levels of LDLc, triglycerides (TG), and total cholesterol (TC).

In women’s volleyball, as in other sports, the first part of the season is a period of heavy training loads that aim to develop technical and tactical skills, as well as achieve adequate physical fitness for the competition period [7]. It is difficult to establish the effects of training on the LP of professional volleyball players. This is because, apart from the personal characteristics of each player, particular features of their training, especially those focused on competition, can substantially modify the LP [8], but we have found no studies that analyse the interaction of these factors. Ruiz et al. [9] commented that volleyball is a sport with a strong component of physical stress, so that playing it leads to lower levels of undesirable plasma lipids and lipoproteins than in the case of other less stressful sports. Witek et al. [10] suggested that changes in the LP over the course of a season could be regarded as transient, with no impact on CVD risk, because the lipid levels remained within normal physiological ranges. Both these studies were, however, conducted in men [9,10].

Thus, the primary aim of this study was to evaluate potential changes in the LP (TG, TC, LDLc, HDLc and atherogenic indices, TC/HDLc and LDLc/HDLc) that might be induced by 11 weeks of training in female volleyball players (FVPs). The secondary aim was to collect baseline data on nutrient intake, in order to advise FVPs from the Spanish Super League concerning the fat content and quality of their diet during this period.

## Methods

The study was designed in compliance with the recommendations for clinical research of the World Medical Association Declaration of Helsinki [11]. The protocol was reviewed and approved by the clinical research ethics committees University of León and the University of Basque Country. The experimental procedures, associated risks, and benefits were explained to eligible players before they gave written informed consent to participate.

### Subjects

The study group consisted of 22 FVPs, undertaking 25 hours per week of performance training (Table 1). All the participants were required to attend the laboratory at two specific points: (a) Day T0 (baseline, prior to their general preparation phase of training); and (b) Day T11 (11 weeks later, after 6 weeks of general preparation and 5 weeks of the specific preparation, as well as 6 matches in the regular women’s volleyball season).

**Table 1:** Example of a week of training

	**Morning**		**Afternoon**	
MONDAY	10:00	STRENGH WORKOUTS All players (1)	18:30	TECH-TAC
TUESDAY	FREE TIME		18:30	TECH-TAC
WEDNESDAY	9:30	STRENGH WORKOUTS Hitters and Libero (2)	15:30	STRENGH WORKOUTS Setters and middle blockers (2)
	10:30	Specific TECH-TAC Setters and middle blockers	18:30	Specific TECH- TAC Hitters and Libero
THURSDAY	FREE TIME		18:30	TECH-TAC
FRIDAY	9:30	13:00	18:30	TECH-TAC
	TECH-TAC	Video		
SATURDAY	OFFICIAL TRAINING		MATCH	
SUNDAY	FREE TIME		FREE TIME	

TECH-TAC: Technical/Tactical training; (1): Basic strength training (maximal strength through hypertrophy); (2): Specific strength training (explosive strength and plyometrics).

All the participating players also completed a diet record to record their food intake during the study and had two sets of anthropometric measurements taken (detailed below).

### Anthropometric measurements

All anthropometric measurements were conducted on Days T0 and T11 by the same Level 2 certified anthropometrist following the protocol of the International Society for the Advancement of Kinanthropometry (ISAK) [12]. Body weight (BW) was measured in kilograms using a SECA^®^ scale, to the nearest 0.1 kg., and height using a stadiometer to the nearest 0.5 cm. Body mass index (BMI) was then calculated using the formula BW/height^2^ (kg/m^2^). A total of six (triceps, abdominal, supra-iliac, sub-scapular, front thigh and calf) skin-fold measurements were taken in millimetres with a Harpenden^®^ skin-fold calliper, to the nearest 0.2 mm and their sum (Σ6SF) calculated. Body Fat mass (FM) was calculated using the Faulkner equation [13].

### Blood collection and analysis

Venous blood samples were drawn after 12 hours of fasting from the ante-cubital fossa of the forearm, between 8.00 and 9.00 a.m. on days T0 and T11. None of the players trained the day before the samples were taken. The TG, TC, and HDLc levels were measured by an enzymatic spectrophotometric technique with an auto-analyser (COBAS FARA; Roche Diagnostics, Basel, Switzerland). These values were then used to calculate the LDLc with the Friedewald equation [14]: LDLc = (TC - HDLc) - TG/5; and the atherogenic indices (TC/HDLc and LDLc/HDLc).

### Dietary control

The participating players were taught how to accurately assess their food intake by dieticians. First, after the T11 anthropometric measurements, the participants where requested to complete a validated food frequency questionnaire (FFQ) for the female Spanish population [15], previously used in other studies conducted in Spain [16,17]. This FFQ, which asked the subjects to recall their average consumption over the previous 11 weeks, included 139 different foods and drinks, arranged by food type and meal pattern. Frequency categories were based on the number of times that items were consumed per day, week or month. Daily consumption in grams was determined by dividing the reported intake by the frequency in days.

Second, as a check on the answers to the FFQ, the participants completed a 7-day dietary record the week prior to starting training (T0) and during week 11 (T11), these questionnaires being distributed on the day the anthropometric measurements were taken. The results obtained by the FFQ were found to be highly reproducible regarding the frequency and amount foods consumed compared to the data from the 7-day dietary records. When it was not possible to weigh food, serving sizes consumed were estimated from either product names, the place of food consumption, standard weights of food items or the portion size indicated in a picture booklet of 500 photographs of foods. Food values were converted into intakes of total energy, fats, different fatty acids, and cholesterol by a validated software package developed by the Spanish Centre for Higher Studies in Nutrition and Dietetics (CESNID), which is based on Spanish tables of food composition [18].

Third, on Day T11, the participants completed a validated 14-point Mediterranean Diet Adherence Screener (MEDAS) [19]. This included 10 items to measure the frequency of consumption of beneficial foods pertaining to the typical Mediterranean diet (virgin olive oil, vegetables, fresh fruits, legumes and pulses, fish, nuts, white meat, and wine in moderate quantities). It also had four items to measure the consumption of foods that should be limited in or eliminated from the diet (red and processed meats; cream, butter, and margarine; carbonated and/or sugary beverages; and commercial bakery products such as cakes or pastries). One point was assigned to each of the 14 items, so that the total MEDAS score ranged from 0 to 14 points, as a continuous measure, and scores above 9 were considered to indicate good adherence to the Mediterranean diet.

### Statistical analysis

All data are reported as means ± standard deviations. Statistical analysis was performed using SPSS, version 19.0 (SPSS, Chicago). A comparison was made of anthropometric characteristics (BW, BMI, Σ6SF, and FM) and their LP parameters (TG, TC, HDLc, and LDLc, as well as the atherogenic indices) on Days T0 and T11, using the Student’s t-test or Mann–Whitney U-test, after normality of the data had been confirmed with the Shapiro-Wilk test. The percentage of change in the outcome variables after 11 weeks was calculated as Δ (%): [(T11 – T0)/T0] × 100. The differences were considered statistically significant when p < 0.05.

## Results

The mean characteristics of the players are summarised in Table 2. Regarding the anthropometric parameters, significant decreases (*p* = 0.027) in ∑6SF were observed over the 11 weeks of the study.

**Table 2:** The anthropometric characteristics of the female volleyball players at T0 and T11 and the percentage changes

	**T0 (n = 22)**	**T11 (n = 22)**	**% Change**	***p *** **T0-T11**
Weight (kg)	69.6 ± 9.4	70.1 ± 9.2	0.8 ± 3.1	0.274
BMI	21.8 ± 2.0	21.9 ± 1.8	0.8 ± 3.1	0.311
Σ6SF (mm)	93.2 ± 26.7	87.5 ± 24.4	-5.2 ± 6.4	**0.027**
Fat mass (kg)	14.3 ± 4.3	13.9 ± 3.9	-2.0 ± 10.1	0.240

Data are expressed as mean ± standard deviation. BMI: body mass index; ∑6SF: Sum of 6 skinfolds.

% Change calculated as: ((T11-T0) x 100/T0).

*p* T0-T11: baseline *vs.* after 11 weeks of training.

The levels of serum lipids and associated indices are listed in Table 3. There were significant decreases in the levels of LDLc (*p* = 0.034), TC/HDLc (*p* = 0.027) and LDLc/HDLc (*p* = 0.030) after the 11 weeks of training.

**Table 3:** The lipid profile in the female volleyball players at T0 and T11 and the percentage changes

		**% Change**	***p *** **T0-T11**
**TG (mg/dL)**			
T0	71 ± 35	0.3 ± 29.3	0.329
T11	65 ± 16		
**TC (mg/dL)**			
T0	182 ± 36	-2.7 ± 15.2	0.284
T11	175 ± 18		
**HDLc (mg/dL)**			
T0	65 ± 16	7.3 ± 22.6	0.089
T11	71 ± 20		
**LDLc (mg/dL)**			
T0	102 ± 38	-7.0 ± 18.1	**0.034**
T11	91 ± 23		
**TC/HDLc**			
T0	3.0 ± 1.0	-9.5 ± 11.4	**0.004**
T11	2.7 ± 0.9		
**LDLc/HDLc**			
T0	1.7 ± 0.9	-13.2 ± 15.4	**0.011**
T11	1.5 ± 0.7		

Data are expressed as mean ± SD. TG: triglycerides; TC: total cholesterol; HDLc: HDL cholesterol; LDLc: LDL cholesterol. % change calculated as: (T11 – T0)/T0 x 100.

*p* T0-T11: baseline *vs.* after 11 weeks of training.

Table 4 compares energy and fat intakes and the recommended allowances for each of these nutrients. Total fat intake, SFA, W6 and cholesterol intakes were above, and MUFAs were below the recommended allowances for adults in the general population, whilst PUFAs and W3 intakes were adequate.

**Table 4:** Energy and macronutrient intake by female volleyball players (n = 22) during the study and the dietary reference recommendations

**Nutrient**	**Per day**	**Per kg BW**	**% total energy**	**Dietary reference recommendations**
Energy (kcal)	2840 ± 268	41 ± 6	100	45-50 g/kg BM/day^a^
Fat (g)	113 ± 20	1.6 ± 0.4	35.6 ± 4.8	15-30%^b^
SFA (g)	35.4 ± 9.8	0.5 ± 0.2	11.1 ± 2.3	< 10%^b^
MUFA (g)	46.9 ± 4.7	0.7 ± 0.1	14.9 ± 2.0	15-20%^b^
PUFA (g)	21.0 ± 7.5	0.3 ± 0.1	6.6 ± 2.0	5-8%^b^
W3 (g)	1.6 ± 0.6	0.04 ± 0.01	0.5 ± 2.0	1-2%^b^
W6 (g)	10.4 ± 3.7	0.4 ± 0.2	4.7 ± 10.0	5-8%^b^
Cholesterol (mg)	443 ± 72	6.6 ± 1.5		< 300 mg/day^b^

Data are expressed as mean ± standard deviation. BW: body weight; SFA: saturated fatty acids; MUFA: monounsaturated fatty acids; PUFA: polyunsaturated fatty acids; W3: omega-3 fatty acids; W6: omega-6 fatty acids; ^a^Recommended energy and carbohydrate intakes [31]; ^b^Recommended lipid intake in the adult population to reduce cardiovascular diseases [2].

With regard to the diet quality of the players (Table 5), the MEDAS score, and W6/W3 and (MUFA + PUFA)/SFA ratios indicated that they consumed a healthy diet, but the MUFA/SFA ratio was below the recommended figure.

**Table 5:** Quality indices for the diet of the female volleyball players (n = 22)

	**Per day**	**Recommended healthy diet**
W6/W3	6.6 ± 6.4	5-10:1^a^
MUFA/SFA	1.4 ± 0.2	≥ 0.5^a^
(MUFA + PUFA)/SFA	1.9 ± 0.4	≥ 2^a^
Mediterranean diet adherence	9.3 ± 2.3	≥ 9^b^

Data are expressed as mean ± standard deviation. SFA: saturated fatty acids; MUFA: monounsaturated fatty acids; PUFA: polyunsaturated fatty acids; W3: omega-3 fatty acids; W6: omega-6 fatty acids. ^a^Recommended healthy diet [41]; ^b^Recommended good Mediterranean diet adherence [19].

Finally, Table 6 shows the daily food intake by the players over the 11-week study and the recommended amounts for the general population and for athletes. Relative to the recommended allowances for athletes, the FVPs consumed smaller quantities of cereals, potatoes, legumes and pulses, and larger amounts of pastries, margarine, fatty meat and cold meats.

**Table 6:** Servings consumed daily by the female volleyball players (n = 22) during the study and the reference recommendations

**Food groups**	**Daily ingested servings**	**Recommended servings ATHLETES**^**a**^
Cereals and potatoes	3.3 ± 0.4	6-11/day
Dairy products	3.1 ± 0.9	3-4/day
Fruits	3.1 ± 0.9	2-4/day
Vegetables	3.8 ± 0.6	3-5/day
Olive oil	1.2 ± 0.4	2-4/day
Other oils	0.3 ± 0.1	Not mentioned
Legumes and pulses	0.5 ± 0.2	2-3/week or frequently (1/day)
Dried fruits	0.4 ± 0.2	2-3/week or frequently (1/day)
Fish	0.9 ± 0.2	2-3/day and alternating these food groups
Lean meats and poultry	1.8 ± 0.4	
Eggs	0.5 ± 0.1	
Fatty meat and cold meats	0.5 ± 0.1	A few times per month
Pastries and margarines	2.1 ± 0.5	
Wine and beer	0.3 ± 0.2	Not mentioned

Data are expressed as mean ± standard deviation of the number of ingested servings for each food group per person per day. ^a^Proposal to adapt the food pyramid to an athlete’s diet [31].

## Discussion

The data collected in this study are of interest because, although the FVPs had a diet rich in fats, cholesterol and SFAs, it was found that their LP did improve. Specifically, LDLc and the atherogenic indices declined, whilst HDLc increased, after 11 weeks of training.

There is strong evidence that aerobic exercise is associated with favourable shifts in blood triglycerides and HDLc; further, data from intervention studies [20] and numerous meta-analyses [21,22] also support the view that there is an LDLc lowering response to exercise training, though this is a less well-characterized and seems to be variable. Furthermore, independent of diet, exercise was found to have beneficial effects on the concentration and size of low-density lipoprotein cholesterol particles, concentration of high-density lipoprotein cholesterol, size of high-density lipoprotein cholesterol particles, and triglycerides [23].

A recent meta-analysis [24] showed that continuous exercise (training) produces a 5 to 8% increase in HDLc levels. This is attributable to an increase in the activity of lecithin-cholesterol acyltransferase (LCAT), which increases the synthesis of HDLc, and a reduction in the activity of hepatic lipase, which is involved in the catabolism of these lipids. The effects of physical activity on LCAT and hepatic lipase depend on the type, intensity, frequency, and duration of the physical activity [25]. Paraoxonases are also associated with HDLc because they induce the hydrolysis of lipid peroxide and they provide protection against atherosclerosis [25]. Additionally, a reduction of up to 20% in paraoxonase levels has been reported in sedentary people [26]. HDLc serum levels are inversely associated with the risk of CVD [8]. In the present study, a slight increase of 7.3 ± 22.6% (*p* > 0.05) was observed in the levels of HDLc in the FVPs after 11 weeks of training. Though the change was not significant, it is interesting to note that an increase of this order of magnitude would decrease their risk of CVD by 16 to 24% [24].

In contrast to HDLc, high levels of LDLc favour the onset and development of CVD [8]. This is why many studies have been conducted to determine which factors lower LDLc levels [6,24,27]. Tambalis et al. [8], in a recent systematic review, pointed out striking evidence that resistance and combined exercise both lower LDLc levels. Likewise, the *Lipid Research Clinics Program*[28] revealed that long-term physical activity, undertaken in a frequent and continuous manner, could decrease LDLc and TC levels. In the FVPs, we observed a slight decrease (by 2.7 ± 15.2%; *p* > 0.05) in TC and a significant decrease (by 7.0 ± 18.1%; *p* = 0.034) in LDLc, changes which add up to an improvement in the LP. The fall in LDLc in the players is attributable to their physical activity having the effect on skeletal muscles of increasing the amount and activity of lipoprotein lipase (LPL). This is an enzyme responsible for hydrolysing TG-rich lipoprotein, thereby reducing VLDL (very low-density lipoprotein) cholesterol and LDLc [29].

Furthermore, it appears that the number of weekly workouts is correlated with increased levels of HDLc and decreased LDLc/HDLc and TC/HDLc atherogenic indices [30]. Specifically, the positive effects of exercise on lipid metabolism were found to last 48 hours [30]. Consistent with this, in our study, the FVPs did two workouts a day, six days a week and significant decreases were observed in their LDLc/HDLc (p = 0.011) and TC/HDLc (*p* = 0.004) indices, of 13.2 ± 15.4 and 9.5 ± 11.4 respectively. Theses decreases in their atherogenic indices can be considered a useful outcome, since high values are strongly associated with the risk of CVD [10].

The daily energy intake of the FVPs during the 11 weeks of study was 41 ± 6 kcal/kg of BW per day. González-Gross et al. [31] advocated an intake of 45 to 50 kcal/kg/day for athletes who train for more than 75 to 90 min/day, as was the case of the FVPs in our study. However, the 39 to 44 kcal/kg/day recommended by Volek et al. [32] for women who engage predominately in resistance exercise training seems more adequate for the first 11 weeks of training in the season in the case of women’s volleyball, because the subjects’ BW remained stable while their FM fell (kg). This was indicated by a significant reduction (*p* = 0.027) in the Σ6SF, skin-fold thicknesses being used as indicators of body FM [33].

It is worth mentioning that total energy intake may also be directly related to the levels of TG, TC, HDLc, and LDLc, especially the amount and type of fat ingested [4]. Fat accounted for 35.5 ± 3.2% of total energy intake by the FVPs, in line with what has been reported by several other authors [34-38], but higher than the data reported by Beals et al. [39] and also higher than the 20 to 35% of the total energy consumed that is recommended for team athletes and for the general adult population [33].

Additionally, the amount of cholesterol and SFA intake was found to be positively correlated with the TC and LDLc [40]. The amount of cholesterol ingested by the FVPs was high (465 ± 57 mg) compared to the 300 mg recommended for the general population [2], similar to the 460 mg reported by Anderson et al. in 15 FVPs at the start of the season after a dietary and nutritional intervention [35] and lower than the 104 mg found by Papadopoulou et al. in teenage FVPs [34]. In addition to these data, we note that the intake of SFAs by the FVPs was also high (11.1 ± 1.2%) compared to the < 10% that has been suggested to be appropriate the general adult population to reduce cardiovascular diseases [2].

This high cholesterol and SFA intake may be due to the players drinking full-fat milk (3.1 ± 0.9 servings/day), even though their daily number of servings was within the recommendations for athletes [31]. In addition, the FVPs consumed relatively large amounts of pastries and butter, foods containing a considerable quantity of SFAs [18], whose consumption is not recommended more often than a few times per month [31] and particularly not more than once daily, as was the case for the players in this study (2.1 ± 0.5 servings/day). For athletes’ nutrition, semi-skimmed or skimmed milk is considered preferable, so as to reduce the intake of cholesterol and calories from SFAs. It is known that the cholesterol metabolism has some negative feedback, in the sense that if large amounts of cholesterol are ingested, the body produces less (in a normal physiological situation). However, an increase in the consumption of SFAs would cause activation of the cholesterol metabolism, with a possible increase in TC [3].

Additionally, the intake of MUFAs (14.3 ± 1.9%) was below the ideal recommended allowance (15 to 20%) [41]. MUFAs have healthy effects on the heart by increasing HDLc levels [5]. It was also established that the ratios between different fatty acids, as measured by the PUFA/SFA (1.4 ± 0.2) and W6/W3 (6.6 ± 6.4) ratios, were within the recommendations (≥ 0.5 and 5–10:1, respectively), while the PUFA + (MUFA/SFA) intake was below the recommended level (1.9 ± 0.4 vs. ≥ 2) for a healthy diet [41].

An inappropriate dietary intake jeopardizes sports performance and the benefits of training. It is crucial to plan a diet education programme to optimise the pattern of food and drink consumed (in this case, increasing the consumption of carbohydrates while decreasing that of fats and proteins) and hence improve athletes’ sporting performance and health.

Future studies should aim to explore LP, as a function of sex, the sport played and the phase of the season (with respect to pre-season, specific preparatory periods, and competitions) and whether there are changes in the profile with diet programmes or supplementation, and in addition should involve hyperlipidaemic subjects.

The limiting factor in this study is the small sample size. For results in future research to be significant, the samples should be larger, or the period of the study should be extended. On the other hand, this study is the first in which the LP of professional sportswomen has been compared with their dietary intake and even these provisional data have allowed us to identify some significant trends that motivate future research.

## Conclusions

According to the data recorded, physical activity during the first 11 weeks of training in the professional women’s volleyball season is heart-healthy because it improves the LP (with a decrease in the LDLc and TC/HDLc and LDLc/HDLc indices). This was true despite the intakes of fats by the players being inadequate, in terms of both quality and quantity. In addition, the exercise carried out by the players during the 11-week study seemed to improve their HDL levels.

## Competing interests

The authors declare that they have no competing interests.

## Authors’ contributions

All authors read and extensively reviewed and contributed to the final manuscript as follows MAJ: Conception and design, analysis and interpretation of the data, drafting and critically reviewing the manuscript. SCP: Interpretation of the data, drafting and critically reviewing the manuscript. UA: Drafting and critically reviewing the manuscript. MSJ: Drafting and critically reviewing the manuscript. SJ: Conception, interpretation of the data, drafting and critically reviewing the manuscript. All authors read and approved the final version of the manuscript.

## References

[B1] Giacosa A Barale R Bavaresco L Gatenby P Gerbi V Janssens J Johnston B Kas K La Vecchia C Mainguet P Cancer prevention in Europe: the Mediterranean diet as a protective choice Eur J Cancer Prev 2013 22 1 90 95 10.1097/CEJ.0b013e328354d2d7 22644232

[B2] Nishida C Uauy R Kumanyika S Shetty P The joint WHO/FAO expert consultation on diet, nutrition and the prevention of chronic diseases: process, product and policy implications Public Health Nutr 2004 7 1A 245 250 14972063 10.1079/phn2003592

[B3] Badimon JJ Santos-Gallego CG Badimon L Importance of HDL cholesterol in atherothrombosis: how did we get here? Where are we going? Rev Esp Cardiol 2010 63 Suppl 2 20 35 20540898 10.1016/s0300-8932(10)70150-0

[B4] Katcher HI Hill AM Lanford JL Yoo JS Kris-Etherton PM Lifestyle approaches and dietary strategies to lower LDL-cholesterol and triglycerides and raise HDL-cholesterol Endocrinol Metab Clin North Am 2009 38 1 45 78 10.1016/j.ecl.2008.11.010 19217512

[B5] Schaefer EJ Lipoproteins, nutrition, and heart disease Am J Clin Nutr 2002 75 2 191 212 11815309 10.1093/ajcn/75.2.191

[B6] Kelley GA Kelley KS Roberts S Haskell W Combined effects of aerobic exercise and diet on lipids and lipoproteins in overweight and obese adults: a meta-analysis J Obes 2012 2012 985902 22523670 10.1155/2012/985902 PMC3317197

[B7] Mielgo-Ayuso J Urdampilleta A Martinez-Sanz JM Seco J Dietary iron intake and deficiency in elite women volleyball players Nutr Hosp 2012 27 5 1592 1597 23478710 10.3305/nh.2012.27.5.5948

[B8] Tambalis K Panagiotakos DB Kavouras SA Sidossis LS Responses of blood lipids to aerobic, resistance, and combined aerobic with resistance exercise training: a systematic review of current evidence Angiology 2009 60 5 614 632 10.1177/0003319708324927 18974201

[B9] Ruiz JR Mesa JL Mingorance I Rodriguez-Cuartero A Castillo MJ Sports requiring stressful physical exertion cause abnormalities in plasma lipid profile Rev Esp Cardiol 2004 57 6 499 506 15225496

[B10] Witek K Changes in serum lipid profile of elite volleyball players in the competition period Biomed Hum Kinet 2009 1 63 66

[B11] World Medical Association Declaration of Helsinki: ethical principles for medical research involving human subjects JAMA 2000 284 23 3043 3045 10.1001/jama.284.23.3043 11122593

[B12] Stewart A Marfell-Jones M Olds T de Ridder H International standards for anthropometric assessment 2011 ISAK: Lower Hutt, New Zealand

[B13] Faulkner J Fall Physiology of swimming and diving Exercise Physiology 1968 New York: Academic press 415 446

[B14] Friedewald WT Levy RI Fredrickson DS Estimation of the concentration of low-density lipoprotein cholesterol in plasma, without use of the preparative ultracentrifuge Clin Chem 1972 18 6 499 502 4337382

[B15] Martin-Moreno JM Boyle P Gorgojo L Maisonneuve P Fernandez-Rodriguez JC Salvini S Willett WC Development and validation of a food frequency questionnaire in Spain Int J Epidemiol 1993 22 3 512 519 10.1093/ije/22.3.512 8359969

[B16] Mariscal-Arcas M Romaguera D Rivas A Feriche B Pons A Tur JA Olea-Serrano F Diet quality of young people in southern Spain evaluated by a Mediterranean adaptation of the Diet Quality Index-International (DQI-I) Br J Nutr 2007 98 6 1267 1273 17640424 10.1017/S0007114507781424

[B17] Bondia-Pons I Mayneris-Perxachs J Serra-Majem L Castellote AI Marine A Lopez-Sabater MC Diet quality of a population sample from coastal north-east Spain evaluated by a Mediterranean adaptation of the diet quality index (DQI) Public Health Nutr 2010 13 1 12 24 10.1017/S1368980009990231 19545468

[B18] Farran A Zamora R Cervera P Tablas de composición de alimentos del CESNID 2003 Madrid: McGraw-Hill-Interamericana

[B19] Schroder H Fito M Estruch R Martinez-Gonzalez MA Corella D Salas-Salvado J Lamuela-Raventos R Ros E Salaverria I Fiol M Lapetra J Vinyoles E Gomez-Gracia E Lahoz C Serra-Majem L Pinto X Ruiz-Gutierrez V Covas MI A short screener is valid for assessing Mediterranean diet adherence among older Spanish men and women J Nutr 2011 141 6 1140 1145 10.3945/jn.110.135566 21508208

[B20] Ronnemaa T Marniemi J Puukka P Kuusi T Effects of long-term physical exercise on serum lipids, lipoproteins and lipid metabolizing enzymes in type 2 (non-insulin-dependent) diabetic patients Diab Res 1988 7 2 79 84 3396267

[B21] Halbert JA Silagy CA Finucane P Withers RT Hamdorf PA Exercise training and blood lipids in hyperlipidemic and normolipidemic adults: a meta-analysis of randomized, controlled trials Eur J Clin Nutr 1999 53 7 514 522 10.1038/sj.ejcn.1600784 10452405

[B22] Kelley G Kelley K Effects of aerobic exercise on lipids and lipoproteins in adults with type 2 diabetes: a meta-analysis of randomized-controlled trials Public Health 2007 121 9 643 655 10.1016/j.puhe.2007.02.014 17544042 PMC1993837

[B23] Huffman KM Hawk VH Henes ST Ocampo CI Orenduff MC Slentz CA Johnson JL Houmard JA Samsa GP Kraus WE Bales CW Exercise effects on lipids in persons with varying dietary patterns-does diet matter if they exercise? Responses in Studies of a Targeted Risk Reduction Intervention through Defined Exercise I Am Heart J 2012 164 1 117 124 10.1016/j.ahj.2012.04.014 22795291 PMC3399760

[B24] Pattyn N Cornelissen VA Eshghi SR Vanhees L The effect of exercise on the cardiovascular risk factors constituting the metabolic syndrome: a meta-analysis of controlled trials Sports Med 2013 43 2 121 133 10.1007/s40279-012-0003-z 23329606 PMC3693431

[B25] Berg A Frey I Baumstark MW Halle M Keul J Physical activity and lipoprotein lipid disorders Sports Med 1994 17 1 6 21 10.2165/00007256-199417010-00002 8153500

[B26] Cabrera de Leon A Rodriguez-Perez Mdel C Rodriguez-Benjumeda LM Ania-Lafuente B Brito-Diaz B Muros de Fuentes M Almeida-Gonzalez D Batista-Medina M Aguirre-Jaime A Sedentary lifestyle: physical activity duration versus percentage of energy expenditure Rev Esp Cardiol 2007 60 3 244 250 10.1157/13100275 17394869

[B27] Stefanutti C Mazza F Multiple lipid-lowering treatment in pediatric patients with hyperlipidemia Med Chem 2012 8 6 1171 1181 22762160 10.2174/1573406411208061171

[B28] Green PP Namboodiri KK Hannan P Martin J Owen AR Chase GA Kaplan EB Williams L Elston RC The Collaborative Lipid Research Clinics Program Family Study. III. Transformations and covariate adjustments of lipid and lipoprotein levels Am J Epidemiol 1984 119 6 959 974 6731434 10.1093/oxfordjournals.aje.a113817

[B29] Kiens B Skeletal muscle lipid metabolism in exercise and insulin resistance Physiol Rev 2006 86 1 205 243 10.1152/physrev.00023.2004 16371598

[B30] Boraita A La práctica deportiva mejora el perfil lipídico plasmático, pero ¿a cualquier intensidad? Rev Esp Cardiol 2004 57 6 495 498 15225495

[B31] Gonzalez-Gross M Gutierrez A Mesa JL Ruiz-Ruiz J Castillo MJ Nutrition in the sport practice: adaptation of the food guide pyramid to the characteristics of athletes diet Arch Latinoam Nutr 2001 51 4 321 331 12012556

[B32] Volek JS Forsythe CE Kraemer WJ Nutritional aspects of women strength athletes Br J Sports Med 2006 40 9 742 748 10.1136/bjsm.2004.016709 16855068 PMC2564387

[B33] Rodriguez NR Di Marco NM Langley S American Dietetic Association, Dietitians of Canada, American College of Sports Medicine American College of Sports Medicine position stand. Nutrition and athletic performance Med Sci Sports Exerc 2009 41 3 709 731 10.1249/MSS.0b013e31890eb86 19225360

[B34] Papadopoulou SK Papadopoulou SD Gallos GK Macro- and micro-nutrient intake of adolescent Greek female volleyball players Int J Sport Nutr Exerc Metab 2002 12 1 73 80 11993624 10.1123/ijsnem.12.1.73

[B35] Anderson DE The impact of feedback on dietary intake and body composition of college women volleyball players over a competitive season J Strength Cond Res 2010 24 8 2220 2226 10.1519/JSC.0b013e3181def6b9 20634750

[B36] Papadopoulou SK Papadopoulou SD Nutritional status of top team-sport athletes according to body fat Nutrition & Food Science 2010 40 1 64 73 10.1108/00346651011015935

[B37] Gabbett T Georgieff B Physiological and anthropometric characteristics of Australian junior national, state, and novice volleyball players J Strength Cond Res 2007 21 3 902 908 17685708 10.1519/R-20616.1

[B38] Hassapidou MN Manstrantoni A Dietary intakes of elite female athletes in Greece J Hum Nutr Diet 2001 14 5 391 396 10.1046/j.1365-277X.2001.00307.x 11906580

[B39] Beals KA Eating behaviors, nutritional status, and menstrual function in elite female adolescent volleyball players J Am Diet Assoc 2002 102 9 1293 1296 10.1016/S0002-8223(02)90285-3 12792630

[B40] Mensink RP Zock PL Kester AD Katan MB Effects of dietary fatty acids and carbohydrates on the ratio of serum total to HDL cholesterol and on serum lipids and apolipoproteins: a meta-analysis of 60 controlled trials Am J Clin Nutr 2003 77 5 1146 1155 12716665 10.1093/ajcn/77.5.1146

[B41] Moreiras Tuni O Carbajal Azcona Á Cabrera L Tablas de composición de alimentos 2005 Madrid: Ediciones Pirámide

